# There is no one size fits all: Elements of implementing virtual bike rides to address loneliness in people living with dementia

**DOI:** 10.1177/20552076241277886

**Published:** 2024-09-26

**Authors:** Kübra Beliz Budak, Pascale Heins., Franziska Laporte Uribe, Simone Anna Felding, Martina Roes

**Affiliations:** 1 German Center for Neurodegenerative Diseases ( 172279 DZNE), Witten, Germany; 2 Faculty of Health, Department of Nursing Science, 5211 Witten/Herdecke University, Witten, Germany; 3 Alzheimer Centrum Limburg, Department of Psychiatry and Neuropsychology, 5211 Maastricht University, Maastricht, The Netherlands

**Keywords:** Dementia, loneliness, active assisted living technology, exergaming, virtual cycling, implementation, long term care, nursing home

## Abstract

**Objective:**

This study aimed to identify factors that hinder or facilitate the implementation of an exergaming technology, SilverFit Mile, which offers virtual cycling, for nursing home residents with dementia in and its potential impact on feelings of loneliness.

**Methods:**

The study followed a descriptive qualitative approach using semi structured interviews with eight care professionals in nursing homes in the Netherlands and based on the Consolidated Framework for Implementation Research (CFIR). Thematic text analysis was used to analyze the interviews.

**Results:**

We identified three main themes and twelve subthemes based on the CFIR. The main themes were residents’ personal characteristics, implementation factors, and loneliness. SilverFit Mile was more suitable for those familiar with cycling and those who enjoyed more solitary activities. Organizational factors such as staff's low digital literacy, lack of time, and need for training were found barriers to implementation, while facilitators included fostering social interaction.

**Conclusions:**

SilverFit Mile was considered positively by care staff based on observations of persons living with dementia. We identified loneliness as a relevant outcome of SilverFit Mile implementation. We argue that SilverFit Mile can foster social interaction between residents and staff through reminiscence or the physical aspect of cycling. However, a better understanding of the connection between loneliness and the use of SilverFit Mile is needed. Overall, our research provides initial ideas about how exergaming technology might address loneliness in dementia.

## Background

Loneliness is identified as an important risk factor for developing dementia.^1–3^ It has been shown to increase the risk of dementia even when controlling for typical risk factors such as depression,^1^ diabetes,^4^ and physical inactivity.^5^ Loneliness is defined as a distressing feeling that accompanies the perception that one's social needs are not being met by the quantity or especially the quality of one's relationships.^6^ It is notably different from social isolation, which refers to being physically away from others.^6^

Persons living with dementia (PlwDs) can experience elevated feelings of loneliness due to the symptoms of dementia, such as declines in cognitive and social functioning^7^ and decreasing social networks due to loss of friends and family.^8^ This in turn was found to be associated with depressive symptoms, lower likelihood of exercise, dysregulated stress response,^3^ and decrease in visuospatial abilities,^7^ which exacerbates loneliness in PlwD.^3^ New research suggests that the relationship between loneliness and dementia could be mediated by hearing and visual impairment, as well as depressive and psychotic symptoms.^9^ Loneliness itself might be the reason behind the reduction in sensory and cognitive stimulation.^9^ This phenomena was found associated with a decrease in neural reserve, which in turn can lead to impaired social cognition.^9^

Loneliness can be exacerbated further if a PlwD moves into a nursing home facility.^8, 10^ For the purposes of this study, nursing home facilities were defined as any established setting providing long-term care to older adults in a residential care facility and/or nursing home. While it is difficult to estimate the proportions of PlwDs living in nursing home facilities in Europe, the percentage of people with dementia living at home ranges from 66% in high-income countries to 94% in low- to middle-income countries,^11^ while global estimates show that the percentage of persons with dementia in nursing homes is more than 80%.^12^

Loneliness among older adults is often addressed through several psychosocial interventions, e.g., telephone befriending or horticultural therapy.^13^ However, despite the increasing number of people diagnosed with dementia each year (WHO, 2017), loneliness interventions fail to directly address PlwDs due to the lack of a comprehensive understanding of the complexity of loneliness and a failure to target the needs and preferences of the residents living in a nursing home.^14^ Therefore, researchers have been considering novel ways to address loneliness in PlwDs, and one of those ways is through the use of technology.

Technology targeted at supporting PlwDs is typically called active assisted living (AAL) technology.^15^ Examples of AAL technology encompass a wide range of technological applications with potential application to dementia care. These include self-contained devices (e.g., tablets, wearables, personal care robots, etc.) and distributed systems (e.g., smart homes, integrated sensor systems, mobile platforms, etc.), as well as software applications (e.g., mobile or web-based apps)^16^ and exergaming devices.^17^ Exergaming technology is described as interactive exercise-based games whereby players engage in physical and cognitive activities.^18^ Exergaming has received more attention in recent years. The literature suggests improved mood and social facilitation as the main benefits of exergaming, as well as overcoming barriers to physical activity, which is linked to better physical fitness, cognition, daily functioning, general health, and well-being.^19^

In our scoping review,^20^ we identified two types of AAL technology that could potentially be successful in addressing loneliness: social robots and multimedia computer systems. We found that loneliness is seldom studied as a primary outcome measure of psychosocial interventions delivered via AAL technology for PlwDs in the nursing home. We concluded that assistive technology holds the potential to impact loneliness in PlwDs living in the nursing home.

With the outbreak of the COVID-19 pandemic in March 2020, there has been a growing interest in loneliness as a phenomenon observed in the context of social isolation and in the use of technology to overcome the frequent gap in many settings caused by a discontinuation of in-person delivered support.^21^ Whether living at home or in a nursing home, worldwide people living with dementia experienced strict isolation regulations in order to reduce the spread of the Corona-virus. Consequently, residents of nursing homes experienced social isolation, loneliness, and a decline in mental health.^22^ Although the use of digital technologies including AAL technology increased among PlwD during the pandemic, the uptake remained low in both home and nursing home settings.^23^ Knowing the pandemic accelerated the use of technology,^23^ there are still many barriers to consider for the widespread adoption and implementation of technology among PlwD. Particularly for exergaming, in the literature an array of challenges has been identified, such as perceived barriers to exercise,^24^ lack of space,^25^ lack of staff member's time,^25^ staff member's low technology competence,^25^ a low belief that the technology will be suited to successfully perform the desired task,^18^ the operational costs,^18^ and the risk of harm when implementing exergaming in the nursing home.^18^ Such risks for users of exergaming technology include falls^18^ and infection.^18^ At the same time, promising links have been observed between exergaming and social isolation and mood, which are related to loneliness.^18, 19^

**Figure 1.**
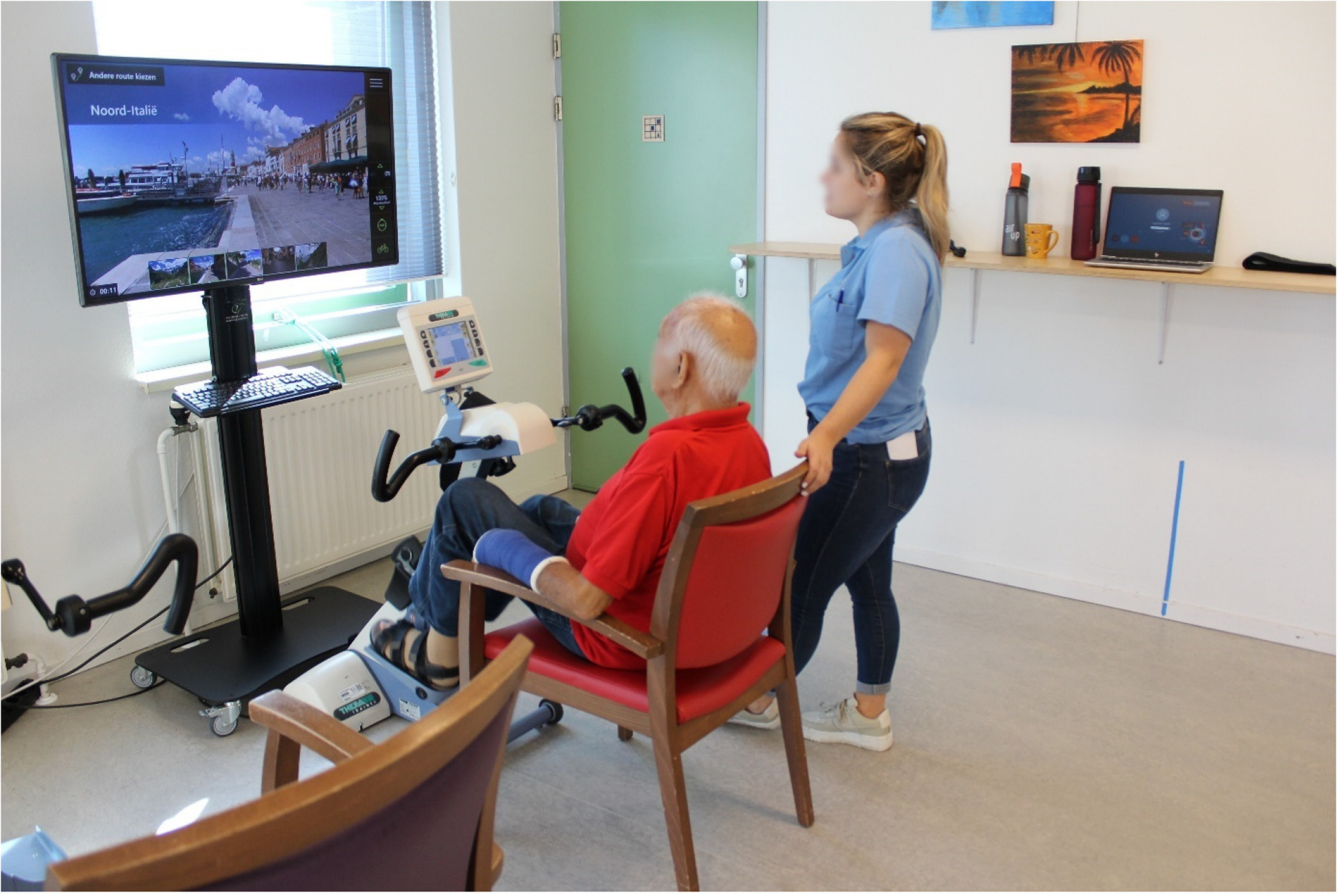
A typical setup for the SilverFit Mile.

Source: SilverFit Website. With permission from SilverFit, copyright owner for this picture.^17^

This study aimed to identify influencing factors that hinder or facilitate the implementation of a specific exergaming technology called SilverFit Mile for residents with dementia in the nursing home and the potential impact of such technology on feelings of loneliness. SilverFit Mile is an exercise bike with a screen in front that displays a point-of-view video of someone cycling in different locations in the world (Figure 1).

Specifically, we were interested in answering the following research question:

1. What are the experiences of care professionals regarding the implementation of SilverFit Mile to address loneliness in the nursing home facilities in The Netherlands?
  - How do care professionals describe their experiences in relation to the acceptance and adoption of SilverFit Mile to address loneliness in PlwDs?
  - Which factors facilitate and hinder the acceptance and adoption of SilverFit Mile?

## Methods

### Study design and setting

The present study followed a descriptive qualitative approach using semi structured interviews conducted with care professionals in the nursing home facilities in the Netherlands. A descriptive qualitative approach allowed the authors to gain an in-depth understanding of the topic of study.^26^ Furthermore, a descriptive qualitative approach gave the authors flexibility when designing and conducting a study.^27^ For the reporting of our interviews and to ensure rigor, we used the Consolidated Criteria for Reporting Qualitative research (COREQ)^28^: A 32-item checklist for interviews and focus groups (Table S1 in the online supplemental materials). This study has received ethical approval from the University of Witten/Herdecke with approval number: SR-205/2021.

### Reflexivity

The interviews were conducted by the first and second author in English. One challenge in this research was that interviews were conducted in English, which was the second language of both the authors and the participants. Furthermore, the authors contacted interviewees through SilverFit. This may have caused some interviewees to believe that the authors were working for SilverFit. In some instances, the first author felt the need to explain to the interviewees that the authors were not working for SilverFit and, in fact, that the interviewees’ personal information would be kept confidential throughout the process of publication. Both first and second authors are cis-gendered white women, although first author comes from a non-European background, whereas second author comes from a European background. Most interviewees were native Dutch, except one which came from another European country. Also, authors did not notice a significant conflict in the rapport between the interviewees and themselves during interviews.

### Analytical frame

**Figure 2.**
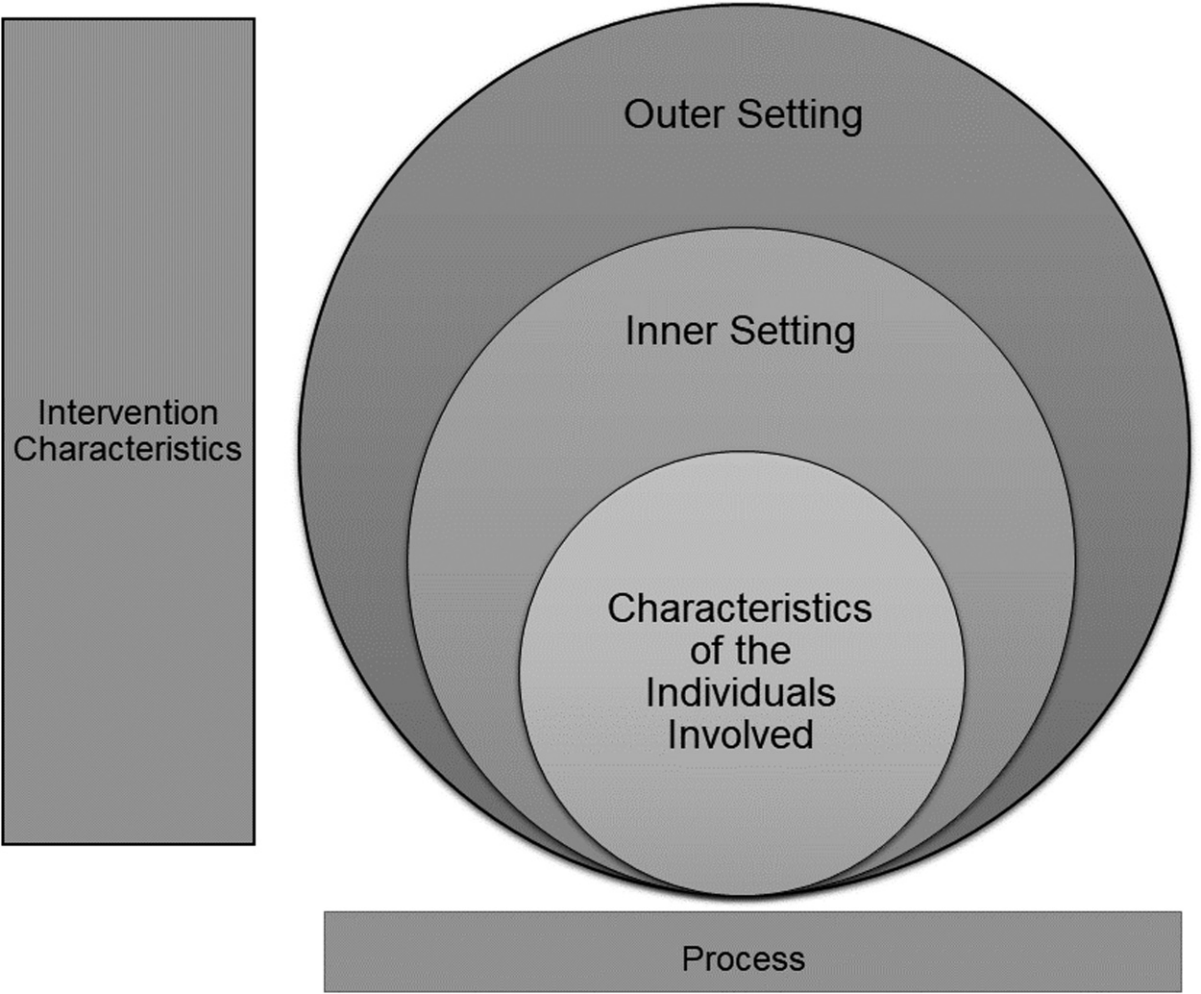
CFIR construct map.

Source: Authors, based on Damschröder et al.^30^

It is recommended to use an implementation framework to guide the exploration of factors that can affect implementation.^29^ The Consolidated Framework for Implementation Research (CFIR)^29^ was used to guide the research process, identify implementation barriers and facilitators, and construct the interview questions. The CFIR provides a pragmatic structure for identifying potential influences on implementation at multiple levels. The CFIR includes 39 constructs (i.e., determinants), organized into five domains: Innovation Characteristics (e.g., complexity, strength of the evidence), Outer Setting (e.g., external policy and incentives), Inner Setting (e.g., organizational culture, the extent to which leaders are engaged), Characteristics of the Individuals Involved (e.g., self-efficacy using AAL technology in a sustainable way), and Process (e.g., planning and engaging key stakeholders). All constructs interact to affect the process of implementation. Therefore, employing this framework enabled the identified barriers and facilitators to be presented in a structured and systematic manner. It also allowed the findings to be easily compared to those of other implementation studies to identify research gaps. We used the original CFIR to guide this paper^29^ (Figure 2) because the updated version^30^ had not been published when this study was planned.

### Recruitment and sample

Participants were recruited through SilverFit (NL), a partner of the EU-funded DISTINCT project. The authors were given contact data through SilverFit about the nursing home facilities that had recently acquired SilverFit Mile. The second author contacted the nursing home facilities individually by telephone and arranged appointments for the interviews. Out of the many attempts to reach approximately 100 nursing homes on the list, 20 of them were reachable. Others were unreachable due to personnel change and contact information change. Twenty nursing home facilities contacted via telephone and/or email, eight care professionals from six nursing home facilities agreed to perform an interview. Twelve participants refused to perform an interview due to sickness, increased workload due to COVID-19 pandemic and lack of time.

In total, two interviews were conducted with two participants that were working in the same nursing home facility, and four interviews were conducted with one participant each. The interviews were conducted in the nursing home facilities in different cities in the Netherlands between February and May 2022. All nursing home facilities included had residents with dementia, but other than one, none had dementia specific units.

### Data collection

The interviews were conducted in English by the first and second authors in a place of the interviewees’ choosing. Other than one occasion, all interviews were conducted in the interviewees’ workplaces in a private room for only the researchers and the interviewee(s). One interview was conducted in a public library meeting space, in a semiprivate place away from other library-goers.

An interview guide based on the CFIR supplemented material^29^ was developed according to the themes identified in our scoping review^20^ and online survey with European Alzheimer associations.^32^ First, the authors studied the CFIR and developed a first draft of the questionnaire. Second, authors pre-tested the questionnaire with three Alzheimer associations who agreed to participate. Third, the authors sought the feedback of the members of the European Working Group of People with Dementia (EWGPWD) in a virtual meeting. The EWGPWD was launched by Alzheimer Europe in 2012 and is composed entirely of people living with dementia, nominated by national dementia organizations of their respective countries.^31^ The EWGPWD works to ensure that the activities, projects and meetings of Alzheimer Europe duly reflect the priorities and views of people living with dementia. Fourth, the authors updated the interview guide (final version), keeping in mind the potential interviewees, their education, and their professional background, and conducted the interviews in English, which was not their first language.

### Data analysis

The interviews were audio recorded and transcribed verbatim by a professional transcription service. Authors did not return the transcripts to interviewees for feedback. No field notes were taken during the interviews. We used thematic qualitative analysis^33, 34^ to better understand the perspective of the care professionals on the implementation process of SilverFit Mile and its possible impact on the feelings of loneliness of the resident. Then, the first and second authors simultaneously went through the data, identifying the emerging themes inductively. Categories and subcategories were initially created independently by the first and second authors and then discussed in an iterative process with all authors using MAXQDA.^35^ All chosen quotes are displayed with an anonymized key such as “(I3AM125-129).” As more interviews were analyzed, more common themes were identified and formed the basis of this paper. All main categories identified are linked to the analytical CFIR^29^ framework. The method for choosing the included CFIR domains was based on the results of our scoping review^20^ and survey.^32^ In those studies, the authors identified the outcomes of technology implementation relevant to the individuals involved in the implementation, e.g., loneliness,^20^ and facilitators and barriers to implementing AAL technology addressing loneliness that were related to the facility, infrastructure, financial issues, and stakeholders, e.g., attitudes of stakeholders. This in turn guided the selection of the themes for the interview guide and thus guided the themes in this paper.

## Results

Six interviews were conducted with eight care professionals in six different nursing homes in the Netherlands, ranging from 39 to 68 min. Table 1 shows the summary of participant demographics. Most participants were females (*n* = 6), and all were activity directors but one participant, who was a physiotherapist. Participants had different levels of experience working with PlwDs (see Table 1).

**Table 1.** Participant demographics.

Demographics	*N*	Demographics	*N*
Role		Experience in dementia care	
Activity director	7	<1 year	1
Physiotherapist	1	Between 1–3 years	3
Age group		Between 4–6 years	0
18–30	3	Between 7–9 years	0
31–50	5	>10 years	3
50+	0	No information	1
Sex			
Female	6		
Male	2		

### Main themes and subthemes

**Figure 3.**
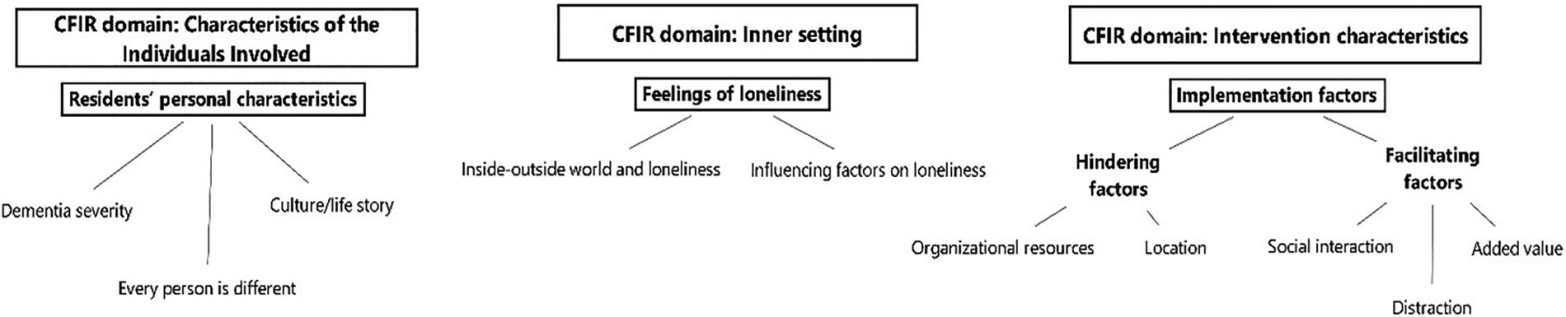
CFIR domains based on 26, presenting main themes and subthemes.

The authors identified three main domains of the CFIR^29^ (see Figure 1): characteristics of the individuals involved, inner setting, and characteristics of the intervention. For each of the three CFIR domains, the authors identified themes and subthemes inductively. In total, three themes and twelve subthemes were identified (see Figure 3).

**Figure 4.**
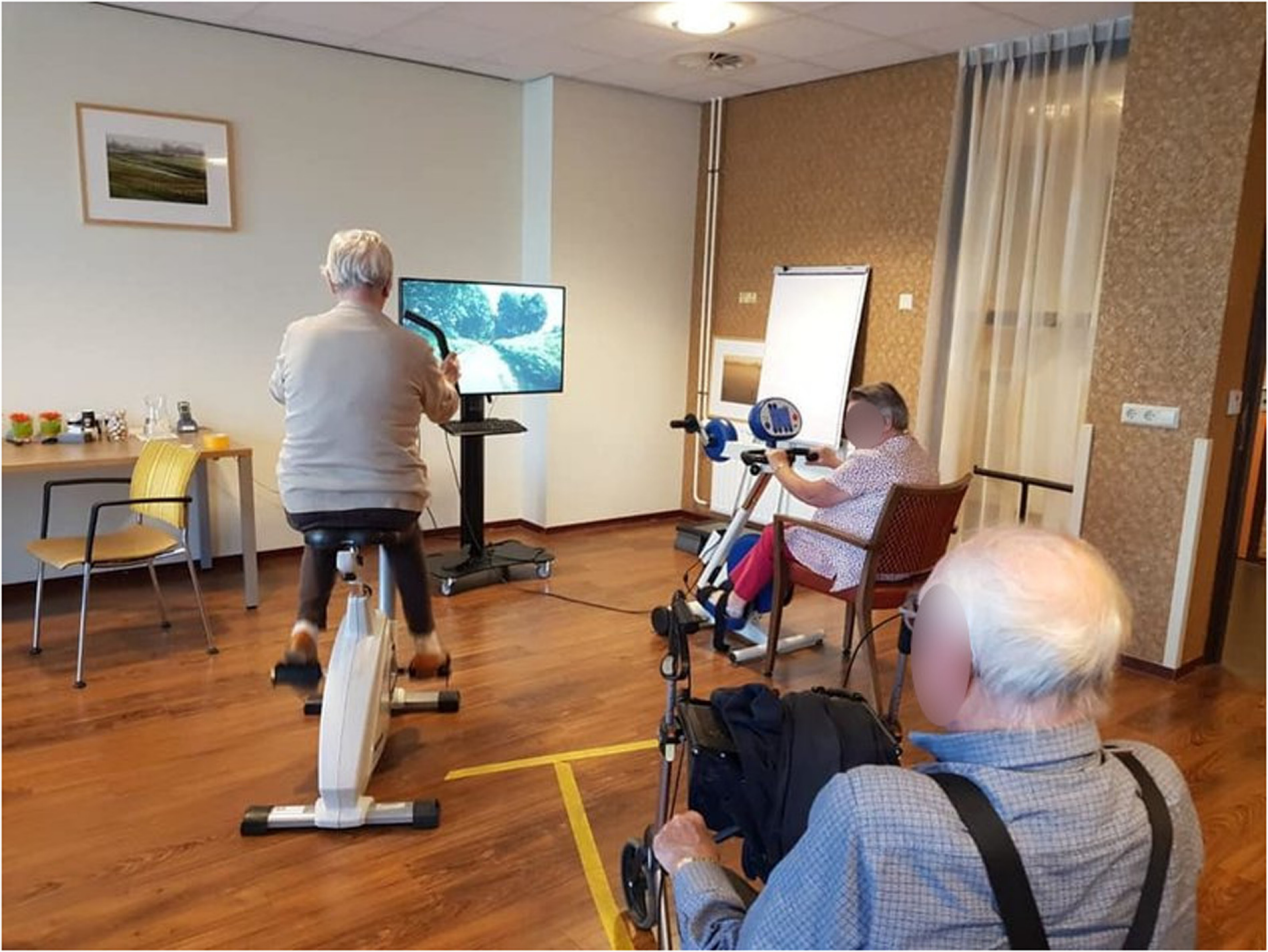
SilverFit Mile can be used by people with disabilities.

Source: SilverFit Website. With permission from SilverFit, copyright owner for this picture.^17^

#### CFIR domain: Characteristics of the individuals

##### Theme 1: Residents’ personal characteristics

###### Subtheme 1.1: Dementia severity

The interviewees often linked the severity of dementia to the acceptance of SilverFit Mile as a possible mediating factor. From their perspective, there seemed to be a dependency between the resident's needs, the use of SilverFit Mile, and the degree of severity of the resident's dementia.

The form of dementia will make the difference of which way it suits more. (…) You have to assess it for the person only because there's no one size fits all. (I5AM253-264)

The residents with more advanced dementia seemed to accept SilverFit Mile more easily and benefit from SilverFit Mile more than others with less advanced dementia. The interviewees pointed out that they showed less resistance to using SilverFit Mile than residents with earlier stage dementia. One staff member argued that this was because every time a resident with advanced dementia was offered to use SilverFit Mile, it was perceived as a new experience and therefore more exciting.

(…) Advanced stage of dementia, I think they would accept a little bit easier. Because any kind of action it's like, okay, let's do it. [They say] “I never did it” even though they did [it] like a half an hour ago (…). [Then] They would say yes, more gladly than these people who are thinking a little bit healthier, let's say like that (…). (I4P605-619)

SilverFit Mile can be programmed so that the pedals can move without the resident using their own legs. This function is often used for people with advanced dementia. Although seemingly useful, the combination of their automatic leg movement by SilverFit Mile and the screen changing its visual scene while they were cycling sometimes seemed to confuse the residents with advanced dementia.

But some people who are further in the dementia and they don't know what you're doing with their legs sometimes, get a bit scared. Or they don't understand the moving in connection to looking at the screen, they still look around. They're biking, but they don't understand the connection that if I'm biking, something is happening over there, maybe that goes too far for some people, but that's it. (I3AM125-129)

###### Subtheme 1.2: Every person is different

The interviewees pointed out that SilverFit Mile appeared to be more suitable for people who did not enjoy group activities. Residents who felt uncomfortable and overwhelmed with group activities seemed to enjoy SilverFit Mile better. Furthermore, the care professionals observed that using SilverFit Mile by themselves and not as a group seemed to calm residents. Thus, the care professionals employed a different approach for each resident suited to their needs and preferences.

But there are also people who are more comfortable alone or very sensitive to any kind of stimulation from outside, or if they’re in a group, they go completely crazy. So, it very much depends because for the people who are more prone to individual therapy, I would gladly grab the SilverFit Mile over the group interventions. (I1AM176-183)

(…) And there are also people who are a little bit on their own. (…) And I think for that kind of people, the SilverFit is better. Because if they don’t like to be as much in a group as other people, SilverFit is better for them. (I5AM275-289)

###### Subtheme 1.3: Culture/life story

From the perspective of the interviewees, residents’ cultural background played a role in their acceptance of SilverFit Mile. Familiarity with cycling as a cultural component seemed to be a critical factor for benefiting from SilverFit Mile. The interviewees concluded that residents from cultural backgrounds without a strong affinity with cycling may not have benefited from SilverFit Mile as much as other residents.

(…) But actually, the Dutch because they’re used to cycling all day and I think they enjoy it more. Because I once went with someone who's an Indonesian lady, and she's just cycling and, and thinking like “Can I get away? Can I get away?” Because yeah, she isn’t used to it. And the Dutch people are used to cycling. (…). (I5AM192-198)

On the other hand, when working with residents with an immigration background, SilverFit Mile can be useful since it can be programmed to show the streets of the resident's location of origin, which they are no longer able to visit due to disease progression. Residents seemed to enjoy the experience of virtually visiting locations they once knew.

(…) Especially with more foreign people who used to live in other countries and immigrated here or who used to travel a lot, that's things that they can’t do anymore. And using the SilverFit Mile, I can bring back those memories and bring back the times where they were freer to go there. (I1AM112-123)

#### CFIR domain: Inner setting

##### Theme 2: Feelings of loneliness

###### Subtheme 2.1: Inside-outside world and loneliness

From the perspective of the interviewees, it seemed that SilverFit Mile helped with feelings of loneliness by showing residents an “outside world”, a world they were no longer able to see in reality.

Most of the people in our care homes are there every day, every single week, every single month of the year. Because they can’t really go out, at least not by themselves, and sometimes they have family to take them places. (…) So, the places that they used to know they cannot go. (I1AM112-123)

Reminiscence is typically defined as a remembered experience, an account of a memorable experience. Seeing the streets that they lived on for many years provided residents with the opportunity to reminisce about their memories and to open up, and it stimulated communication. Talking about their memories and seeing places they had not seen in a long time seemed to elevate residents’ mood.

I have a Portuguese man, there's one route that looks kind of like his hometown in Portugal. And he just completely loves it because he can, he cannot go back there physically (sighs), but he still misses it every day. (…) It's not the same, but he kind of recognizes it uh, makes him more believe that he is at home. (I1AM139-145)

Furthermore, using SilverFit Mile seemed to bridge the gap between residents’ lives before dementia and their lives living with dementia, and it contributed to improving residents’ moods.

It's [SilverFit Mile is] putting them in a situation where they used to be. Like in a comfortable situation where they used to bike and do the normal things. What they used to do before in the past, they can easily go really, really into their like nice memories. (I4P97-108)

The interviewees observed that SilverFit Mile helped create bonds between residents and staff. Doing a physical activity together helped the interviewees connect with residents and seemed to help residents if they were feeling lonely.

It [SilverFit Mile] creates a bond if you do it a lot with people. Het is tegen eenzaamheid en bewegen, dus eigenlijk wat in elkaar verweven. [Dutch for ‘It is against loneliness and moving, so it's actually somewhat intertwined.’] So, yeah, I think initially people don't buy it to prevent loneliness, but later on they see, ‘Hey, this is a really good way to connect people’. (I3AM151)

It appears that with the combined experience of seeing places that they liked or lived and cycling again through those streets *like before*, residents gained a sense of control. The interviewees pointed out that a sense of being in control *once again* may have been linked to the resident's mood and may have motivated them to converse with others. Although loneliness is a subjective feeling and is not necessarily resolved by social interaction, the interviewees reported that in this instance, the experience of cycling with SilverFit Mile through a familiar area could potentially reduce feelings of loneliness.

(…) SilverFit Mile is great in bringing people to places that they haven’t been in a while or just recognizable. (…) And usually, because of paranoia or being scared or just being sad, they get lonely because they do not want to get involved with anything. And I do have a few people who used to say no to everything. But if I come with the bike, they gladly come because it's something that they have grown to love, and every time I get in, they are like, “What route are we gonna cycle today?”, “Where are we gonna go?”. And then uh, they can pick something and feel more in control about what they’re doing and still have a more enjoyable time. (I1AM193-201)

###### Subtheme 2.2: Factors influencing loneliness

Experience of loneliness can be different for people from different backgrounds for example immigrants.

We have a lot of people with different cultures in here and with dementia. (…) So, back then they spoke Dutch very good, now they forget it. So, they cannot talk to other people in their living room. And yeah, that's also a reason for loneliness. (I2AM18-18)

Randomly remembering people or things that they lost causes emotional distress to PlwDs. Forgetting visits by family and residents with immigrant backgrounds who lose the ability to talk in the second language are factors that may contribute to feelings of loneliness. Loneliness is already a very personal phenomenon, and dementia symptoms seem to complicate the matter even more and elevate feelings of loneliness.

(…) And I do see that people with dementia are more prone to loneliness simply because they stop understanding things. They do not understand what's happening around them. Their environment can be very strange and sometimes threatening to people. They have more problems trusting other people […]. So, a lot of people are very, very sad about things that happened like a long time ago. (I1AM65-75)

#### CFIR domain: Intervention characteristics

##### Theme 3: Implementation factors

This theme has been split into facilitating and hindering factors that influence implementation.

###### Subtheme 3.1: Facilitating factors

This subtheme consists of different influencing factors: (a) social interaction, (b) distraction, and (c) added value.

- *Social interaction:* The interviewees observed that SilverFit Mile facilitated social interaction by acting as something to talk about. They mentioned that SilverFit Mile facilitated social interaction by being a conversation topic among residents and staff. In regard to the interaction between residents and staff, SilverFit Mile appeared to foster conversation due to the videos on screen. Talking about the places viewed on screen fostered social interaction, kept the conversation going, filled lulls in the conversations and motivated residents to open up and talk more easily than usual.(…) And a lot of the people who are very closed off and very insecure, suddenly start telling stories when they use the SilverFit Mile because they recognize things that they haven’t seen in a long time or sometimes even from their youth, which they do not really remember and then they completely open up and blossom, with the view of what they used to know and used to like. (…) And just the fact that they are in a different state of mind because they see different things, their whole behavior can change with that. (I1AM112-123)

The interviewees reported that SilverFit Mile also fostered social interaction between residents. When SilverFit was placed in a common area, other residents would come and join the resident on the SilverFit Mile, who might be accompanied by a staff member. Talking about the SilverFit Mile and the places in the videos would facilitate social interaction, and staff members did not observe that the resident using SilverFit Mile felt uncomfortable due to this interaction.

I think because they're social with me or with somebody else who is passing… SilverFit Mile, it's really next to the lift… So, lots of people are passing and then they're having comments and then they're a little bit proactive in that sense… (I4P132-141)

That is my point of view that bikes should be in a social place where people are passing. So, people are having this extra impact of sitting and doing something and others are making some comments about it saying “lekker bezig” [Dutch for ‘well done’] or something. And then they would respond really nicely. And basically, I think they like any kind of contact. (I4P295-308)

*Distraction*: The care professionals pointed out that they used distractions for residents who appeared distressed because of dementia symptoms. From the perspective of the health care professionals, it seemed that distracting residents was a way to keep their behavior from escalating.But they can have this switch that sometimes, they have a blank and they don't understand like where their family is and why they're not at home (…) And I think the best way is hun af te leiden [Dutch for ‘is to distract them’]. (…) Never say, “No, it's not true”, never go against their story, not trying to make it worse their feeling, when they're sad, they can be sad (…) but eventually, you will try to distract them and make them feel better by maybe doing something together. (I3AM47-51)

This strategy, however, did not seem to be necessarily targeted at making residents feel happier but engaging them in activities such as talking about something else or taking a walk together to prevent worsening of a resident's low mood. Thus, SilverFit Mile could be used to distract residents while communicating with them about something other than what they were worried about. Furthermore, the use of SilverFit Mile stimulated reminiscence about good memories with the added benefit of exercise.

I think it [SilverFit Mile] distracts people when they're in this mood of (…) agitated or sad. It helps them to distract. Distraction helps a lot; whatever activity you do. Second, it's really good to move, it lifts your mood to do something physical. And yeah, it gives you a connection to talk about something that they're seeing. It brings back memories, and it makes a connection with the person who was doing it with them. And conversation starts usually when they see the videos. (I3AM78-82)

*Added Value:* SilverFit Mile is an accessible alternative for exercising. For residents unable to use a conventional exercise bike, SilverFit Mile could be used while sitting on a chair or a wheelchair (Figure 4).

SilverFit Mile seemed to make it easy for many residents to keep doing exercises for a longer time and moving on to more intense exercises.

(…) I think it makes a lot of things easier as well because it's very much a stimulating thing for many people and they do like coming there. So, we usually start with the SilverFit and the bike. And then we move on to like the other exercises that might be harder for them to (do). (I1AM211-221)

SilverFit Mile could also be used for physiotherapy, which was convenient for both the care professionals and residents because they did not have to travel for physiotherapy as often as they had before.

Sometimes, if people used to go to the physiotherapist and with the SilverFit, people don’t have to go (anymore) because they’re moving here. (…). (I5AM447-455)

###### Subtheme 3.2: Hindering factors

This subtheme consists of different influencing factors: (a) organizational resources and (b) location.

- *Organizational resources:* The interviewees pointed out that a lack of proper staff training and low technology competency in staff were key hindering factors when implementing SilverFit Mile in the nursing home. Staff shortages were also reported as a factor. When there are not enough staff, exercise is not a priority.… I think, it [SilverFit Mile] will help, but we can use it more often. Honestly, I'm the only one, together with one of the physiotherapists, who knows how it works. So, when I'm not here and he is not here, nobody is gonna use it. (I2AM30-32)

At the same time, other staff members abstained from using SilverFit Mile, stating concerns about not knowing how to use it, being afraid of breaking it or simply being too busy.

I think, they're always thinking, I don't have time for that, “oh, no, it's technology, I can't work with computers.” Some think, “maybe I do something wrong and then it will break or something.” They don’t know how to use it. So, that's the fear, I think. That they think, “Oh no, I don't know, how to use it. (I2AM141-143)

*Location:* The placement of SilverFit Mile seemed to be linked to the behavior of residents. For example, when SilverFit Mile was located in a public place, it seemed to make some residents feel uncomfortable using SilverFit Mile.We used to have the SilverFit always in a different room, like our therapy room. But for a lot of people, it's pretty hard to leave their more comfortable location and go into the rooms that they do not know can be very intimidating because sometimes there's other people they do not know. (I1AM280-287)

On the other hand, SilverFit Mile facilitated social interaction among residents when it was placed in a public area. At the same time, the care professionals noted that when SilverFit Mile was placed in a private room, it also made residents feel intimidated, which resulted in residents refusing to use SilverFit Mile.

(…) People are like ‘other people see me on the bicycle and I don’t like that.’ It's a kind of privacy or something. It's confronting for them. (IAM6221-238)

## Discussion

In this paper, we aimed to identify factors influencing the implementation of exergaming (here, SilverFit Mile) in the nursing home through the experiences of care professionals, and to understand how using AAL technology might be linked to feelings of loneliness in PlwDs. Therefore, we interviewed care professionals and were able to identify three main themes under the umbrella of three CFIR domains.

### CFIR and loneliness as an outcome

The analysis and this paper were structured according to the CFIR^29^ to systematically present the implementation factors of SilverFit Mile. The CFIR has been updated, and one of the prominent points is the emphasis on anticipated and actual intervention outcomes,^30^ but this updated version was published after we had finished planning the current study. Our data show that loneliness could be considered an actual resident-related outcome of SilverFit Mile implementation and could be linked to the CFIR domain *Networks and Communications.* This CFIR^29^ domain describes the nature and quality of webs of social networks and the nature and quality of formal and informal communications within an organization. Damschroder et al. use the term *social capital* within this domain,^29^ which describes the quality and extent of relationships.^29^ The definition of social capital overlaps with the definition of loneliness used in this paper.^6^ One component of social capital is the internal bonding of individuals within the same organization,^29, 36^ which in our study refers to the relationships among residents as well as the relationship between residents and staff. Social capital and relationship building may act as factors that can potentially address loneliness through the stimulation of reminiscence and social interaction. It can be argued that this study identified a potential link between SilverFit Mile implementation in the nursing home and feelings of loneliness in PlwD.

### CFIR domain: Characteristics of the individuals

The role of dementia itself, specifically in people with advanced dementia, is important to consider. PlwDs may become confused or irritated by SilverFit Mile's automatic exercise function.^37^ We learned in our study that a resident with advanced dementia may feel uncomfortable being strapped down on the pedals with their legs being moved by a machine. This can also be exacerbated if a resident becomes further confused with the connection between the image on the screen and the automatic cycling.^37^ This phenomenon can appear due to neurodegeneration, which can lead to a different idea of reality in the experiences of PlwDs.

Exercise as an intervention in relation to neuropsychiatric symptoms of dementia such as psychosis (delusions and hallucinations), agitation, aggression, depression, anxiety, apathy, motor disturbances, and appetite and eating problems,^38^ has been increasingly investigated in the last years.^39, 40^ Other studies have shown^39, 40^ from little to significant evidence for exercise in general. Exergaming, similarly, provides inconsistent evidence related to neuropsychiatric symptoms of dementia.^19, 41–43^ Some studies provide evidence on the role of dementia severity in moderating neuropsychiatric symptoms of dementia, suggesting that older adults with mild or moderate dementia appeared to benefit from exercise interventions rather than older adults with severe dementia.^42^ This is somewhat contradicting to our results, which are suggesting that older adults with severe dementia might benefit more from exergaming, than others with less severe dementia. It is mainly attributed to the mobility and sensory challenges which often appear parallel to dementia.^42, 44^ However, we obtained our results from a small qualitative sample and the potential benefit is described in terms of acceptability of and joy in using an exergaming tool, whereas Chen et al.^42^ discussed dementia severity as a moderating factor for the favorable outcomes of exercise for neuropsychiatric symptoms. Other studies also suggest that different strategies need to be applied depending on dementia severity, since people experience different symptoms,^39, 43^ which is in line with the results of the current study.

Somehow, cycling itself is a memory they still have and remains relatively intact, but in the present, sitting in front of a screen with one's legs moving on their own feels strange. This might be due to changes in the capacity of spatial and temporal orientation.^45^ Experiencing feelings of loss (not cycling on one's own anymore) may also cause confusion because “dementia threatens the identity and sense of worth and changes their roles and the relationship to others”^46^ and corresponds with feelings of loss of autonomy, control, and connection.

The personality or background of the resident was identified as a relevant factor in the implementation process. Residents who prefer solitary activities to group activities can benefit from using the SilverFit Mile, and vice versa. Although previous research suggests the need for more personalized exergaming experiences,^37, 47^ this topic is seldom investigated. There is a need for inclusive design with PlwDs and their care partners in the implementation process.^48^

The cultural background of the resident can decrease the threshold for residents to use the SilverFit Mile or it can increase the threshold. Although some research suggests better outcomes in cases of loneliness^49^ and social isolation^50^ when exergaming is provided with a culturally familiar component, there is a lack of research on this topic.

### Feelings of loneliness and sense of control

The interviewed staff members pointed out that based on their experience, SilverFit Mile can potentially help decrease feelings of loneliness. Although the exact mechanism of this relationship is beyond the scope of this paper, it can be argued that the combination of reminiscence and social interaction influences feelings of loneliness. Psychosocial intervention studies aimed at loneliness have been considered successful; however, current research has yet to discover the potential of AAL technology.^20^

It is well known that more social interaction does not equal less loneliness and that reminiscing about good times cannot *cure* loneliness.^6^ However, there is evidence that improved social interaction, meaning facilitating deeper, high-quality connections^51^ and engaging in meaningful activities in the nursing home environment,^52^ can potentially help to reduce feelings of loneliness. Our results suggest that social interaction could be fostered by using SilverFit Mile.

The interviewed staff also pointed out that the use of SilverFit Mile potentially helps residents gain a sense of control by providing them control over their use of the exergaming, for example, by choosing the location seen on screen. Research shows that a decreased sense of control over one's life can be a mediating factor between dementia and loneliness via a diminished sense of control over obstacles that lie between one's goals and oneself.^10^ This phenomenon was reported in another study that found that the following four aspects of life in the nursing home were deemed important by PlwDs, their families, and care professionals: sense of personal identity, sense of control, sense of being needed, and sense of worth.^53^ Additionally, in a study by Moyle et al., the nursing home residents with dementia expressed their need for a sense of control over their lives as one of the most important needs in the nursing home.^54^ A lack of control over their daily schedules, where they go, and what they do seems to contribute to residents’ loneliness.^54^ One might argue that choosing a place to cycle that is meaningful for the resident, controlling the speed and therefore the video on the screen, and reminiscing with staff or other residents and thus socializing, can be identified as factors underlying this link.

### CFIR domain: Intervention characteristics

Our second theme revealed facilitating and hindering factors related to the implementation of SilverFit Mile. Facilitating factors include fostering social interaction, providing a distraction, and adding value. SilverFit Mile fosters social interaction between residents, staff, and other residents, confirming previous literature,^37^ and has adjustable features that allow it to address the needs of people with physical disabilities.^37^

An unexpected finding (added value) of our study was that the implementation of SilverFit Mile reduced the need for residents to go to physical therapy. It can be argued that perhaps the technology was especially useful in times such as the COVID-19 pandemic, when the nursing home facilities had to isolate in place.

The COVID-19 pandemic has fast-tracked the adoption of technology in the context of dementia support and care.^23^ For example, in a national survey %72.8 of respondents among German nursing homes had created additional opportunities for care recipients with dementia to use digital technologies in order to facilitate social interaction.^55^ Similarly, a study conducted in three European countries and Australia showed that video calls, and group-chats were effectively used across countries by people living with dementia and their family-carers to maintain relationships with professionals, families, and small groups of peers.^56^ Also, non-pharmacological interventions, such as cognitive stimulation therapy, were delivered virtually instead of in-person.^57^ This increased application of technology to care contexts also revealed barriers to implementation, such as low digital literacy of care staff^55^ or family-carers.^57^ This current study's results suggest that a lack of personnel, insufficient personnel education and staff's low digital literacy were hindering factors. This is in support of findings from the literature, where care professionals’ lack of time due to lack of personnel^58^ and low digital literacy^59^ are commonly identified barriers to the successful implementation of AAL technology in nursing homes. Furthermore, Barbosa et al. found in their rapid review that individuals’ physical, cognitive, or sensory difficulties might negatively affect the adoption of technologies.^23^ They concluded that the adoption and use of technology in the context of dementia support and care use is likely to continue beyond the pandemic and that we need to ensure that people living with dementia are empowered to use it.^23^ The results of this study are in accordance with the literature,^23, 55, 57–59^ which indicates the importance including the needs and preferences of PlwD when implementing a technology. Also, while the increasingly important role of AAL technologies and their potential to address care gaps during crises have been demonstrated during the COVID-19 pandemic, institutions and stakeholders involved in the care of older persons seem often ill-prepared for a crisis as a study has recently found, e.g., for Germany.^60^ There is a need and an opportunity to adapt a cross-country learning approach and, e.g., learn from those countries more experienced in adapting to crises.^61^

There are findings showing that older adults can benefit from distraction, an effect that can be seen when the distraction is congruent with the task that they are performing.^62^ Confirming the literature,^63^ SilverFit Mile is used to distract residents in distress, not necessarily to make them happy, but rather to help them find something they like to do. Nevertheless, there are ethical concerns regarding distraction.

### Distraction and ethical concerns

Ethical implications of distraction have been discussed at length in the literature.^64–66^ Huang and Cong suggest that while distraction is likely to be morally justified in many situations, in dementia care, it is usually not the last resort but a means of convenience.^64^ However, the patient's ability to interact, autonomy and dignity are generally ignored.^64^ That said, morally defensible distraction is considered context-specific and included in the caring process, which requires instant, creative, and interactive care procedures to identify under what circumstances and in what ways a distraction is morally justified.^64^ This understanding of distracting PlwDs highlights the aim of helping PlwDs improve their mood by engaging them in something they enjoy and helping them increase communication with other residents and staff and therefore changing their mood and perhaps feeling less lonely.

The same is argued for technology that is used for distraction. If a technology is used because the caregiver is engaged in other tasks, this comes with certain but low risks.^67^ However, it is also noted that the risk is modified by the PlwD's particular situation and thus must be considered individually.^67^

Technologies may lack the intelligence and empathy required to truly know a person and his or her life history and preferences,^68^ even though these are important factors of implementation. Technologies are typically not sensitive to natural human emotions such as surprise, confusion, anger, or disappointment, and they cannot respond adequately to such emotions.^68^ Importantly, even though care technologies have clear moral implications, they are not moral agents in themselves; they cannot take an “all things considered” approach that is required to support ethical decision making.^68^ Therefore, it is important to realize the importance of care professionals in dementia care and consider the capabilities and risks that technology might bring in the care relationship.

### Private and public locations

A public location of SilverFit Mile in a nursing home can be a concern for some residents, such as those who may find it challenging to use SilverFit Mile if others are watching. This is an unexpected result that has not been reported in previous research, which has suggested that exergaming should be near the public areas so it can be used anytime.^37^ On the other hand, some residents find it overwhelming to use SilverFit Mile if it is in a separate room. However, previous research has suggested that a quieter surrounding has often been better accepted by residents.^37^

Residents’ different reactions to cycling in a public or private place bring other issues to mind, such as the personality, identity, personhood, and privacy of the resident. Privacy is considered an important human need, which can be defined as “a process of boundary control available to be exercised by the individual to manage both personal and social interactions”.^69, 70^ It has been suggested that if the impact of public or private space on older adults is to be understood, personal space and territoriality must be considered.^69, 71^ Privacy regulation mechanisms have been described as verbal and nonverbal behavior and environmental behavior, for example, claiming physical and personal space.^72^ Residents’ preferences regarding where to best place SilverFit Mile can be interpreted as a way of regulating their interpersonal space. Research suggests that PlwDs regard place as an important element of personhood.^58^ Personhood can be defined as “a standing or status that is bestowed upon one human being, by others, in the context of relationship and social being”.^73^ Therefore, personhood and its link to the sense of privacy and public or private spaces must be considered in a person-centered care schedule and residents’ preferences.^74–77^

This paper investigated the implementation factors of SilverFit Mile in six nursing homes in the Netherlands. Care professionals reported the importance of taking the personality, background, and dementia severity of residents into consideration when choosing and implementing an exergaming technology. Reduction of loneliness in PlwDs was an unexpected outcome of SilverFit Mile implementation. Care professionals noted that even though this outcome was unanticipated, SilverFit Mile, by fostering social interaction and reminiscence, can potentially address feelings of loneliness in PlwDs. The main facilitating factors were increased social interaction, the possibility of the technology being used to distract residents if they are in distress and added value, e.g., the possibility of being used for physiotherapy. The main hindering factors were organizational resources, e.g., personnel's lack of time and training and the location of SilverFit Mile, which elicited different reactions from different residents. Overall, SilverFit Mile was considered positively by the care professionals and deemed “the fun of the residents.”

### Strengths and limitations

To our knowledge, this is the only paper that has focused on loneliness in relation to an exergaming technology intervention in the nursing home for people with dementia. Second, this paper reports on the first-hand experiences of care professionals that implemented SilverFit Mile themselves. Third, the paper can be used to guide the use and implementation of exergaming technologies in the nursing home environment. Furthermore, by indicating a potential link between loneliness and exergaming use, this research can be used to guide future research. Finally, by using the liberal descriptive qualitative approach, the current research presents in depth data, untethered by the need for strict labels and definitions.^78^

One limitation of this study is the relatively small number of interviews (*n* = 8). This might have been in relation to a language barrier, with one of the interviewers/first author not speaking Dutch, the project-running time, as well as the restrictions in the context of the COVID-19 pandemic at the time of recruitment, such as, recurrent sick leaves of the staff members and understaffing in nursing homes. However, even with a relatively small sample, all authors were able to ensure data saturation. As suggested in the literature, authors paid attention to the following aspects of the analysis to decide if and when data saturation was reached: sufficiency of information to replicate the study,^79–81^ obtaining new information,^79, 82^ and further coding is no longer feasible.^79, 82^ When all authors were convinced that all these aspects were fulfilled, they continued with their analysis. Furthermore, qualitative interviews have been a popular method in the same field as this study, for an in-depth analysis of the implementation process.^18, 83, 84^

A second limitation is that all interviewees spoke fluent English; however, almost all struggled to find words occasionally and had to refer to the second author, who spoke Dutch. Second, the first author felt the need to occasionally ask clarifying questions during the interview to not rely heavily on the context of the current conversation.

This research is based on the perspective of care professionals in the nursing home, but it is about PlwDs. Thus, the third limitation of this research is that the interviewees were not PlwD themselves but care professionals. Given the language barrier, dementia symptoms and limitations in obtaining consent, it was not possible for this research team to interview PlwDs themselves.

Finally, flexibility or variability in qualitative descriptive methods may have had hindered the comparability and reproducibility of this research to a degree.^78^

## Conclusions

This paper contributes to the literature in two ways: describing the implementation factors of the SilverFit Mile and describing a potential link among SilverFit Mile, loneliness, and sense of control. SilverFit Mile was considered positively by care staff based on their observations with PlwDs, although with limitations. All barriers related to staff were identified as modifiable factors that could be improved by improving support and training.

It may be argued that SilverFit Mile can foster social interactions between residents and staff through reminiscence; however, more research is needed to uncover this potential connection. The combination of reminiscence and social interaction may potentially address loneliness through an improved sense of control, although further research is needed to understand the mechanism. Given the lack of loneliness interventions targeted at PlwDs in the nursing home, further research is needed to identify dementia-specific loneliness interventions.

The location of SilverFit Mile elicited different reactions from different residents, which highlighted the topics of personhood and privacy. Personhood and its link to the sense of privacy and public or private spaces must be considered in a person-centered care schedule. Further research is needed to identify the potential links among personhood, perception of private and public spaces, and loneliness in PlwD.

PlwDs’ need for a sense of control over their lives when living in a nursing home could potentially be linked to loneliness. PlwDs’ desire to have some sense of control over their lives in the nursing home must be acknowledged by care professionals and families. They must be given options regarding their daily schedule and opportunities to socially engage with others in a meaningful way. Care professionals need to consider residents’ personality, severity of dementia, and cultural background when planning activities for and with the resident. This practice must be extended to which technologies are acquired in the first place, because cultural and personal background of a person may prevent them from benefiting from an activity that do not fit their preferences or needs, e.g., cycling. In this case care practitioners may benefit from an open and inclusive communication with the PlwD and their families in order to identify the best course of implementation plan. Exergaming technology such as SilverFit Mile seems to be better suited for those who are culturally familiar with cycling. Future research is needed to uncover the exact mechanism of the relationship among exergaming, loneliness, and sense of control.

## Supplemental Material

sj-pdf-1-dhj-10.1177_20552076241277886 - Supplemental material for There is no one size fits all: Elements of implementing virtual bike rides to address loneliness in people living with dementia Supplemental material, sj-pdf-1-dhj-10.1177_20552076241277886 for There is no one size fits all: Elements of implementing virtual bike rides to address loneliness in people living with dementia by Kübra Beliz Budak, Pascale Heins, Franziska Laporte Uribe, Simone Anna Felding and Martina Roes in DIGITAL HEALTH

sj-pdf-2-dhj-10.1177_20552076241277886 - Supplemental material for There is no one size fits all: Elements of implementing virtual bike rides to address loneliness in people living with dementia Supplemental material, sj-pdf-2-dhj-10.1177_20552076241277886 for There is no one size fits all: Elements of implementing virtual bike rides to address loneliness in people living with dementia by Kübra Beliz Budak, Pascale Heins, Franziska Laporte Uribe, Simone Anna Felding and Martina Roes in DIGITAL HEALTH

sj-docx-3-dhj-10.1177_20552076241277886 - Supplemental material for There is no one size fits all: Elements of implementing virtual bike rides to address loneliness in people living with dementia Supplemental material, sj-docx-3-dhj-10.1177_20552076241277886 for There is no one size fits all: Elements of implementing virtual bike rides to address loneliness in people living with dementia by Kübra Beliz Budak, Pascale Heins, Franziska Laporte Uribe, Simone Anna Felding and Martina Roes in DIGITAL HEALTH

sj-docx-4-dhj-10.1177_20552076241277886 - Supplemental material for There is no one size fits all: Elements of implementing virtual bike rides to address loneliness in people living with dementia Supplemental material, sj-docx-4-dhj-10.1177_20552076241277886 for There is no one size fits all: Elements of implementing virtual bike rides to address loneliness in people living with dementia by Kübra Beliz Budak, Pascale Heins, Franziska Laporte Uribe, Simone Anna Felding and Martina Roes in DIGITAL HEALTH
