# Supplementary material for: There is no one size fits all: Elements of implementing virtual bike rides to address loneliness in people living with dementia
Source: Digit Health. 2024 Sep 26;10:20552076241277886. doi: 10.1177/20552076241277886 (PMC11437561; doi:10.1177/20552076241277886)
Supplement: sj-docx-3-dhj-10.1177_20552076241277886 - Supplemental material for There is no one size fits all: Elements of implementing virtual bike rides to address loneliness in people living with dementia [file sj-docx-3-dhj-10.1177_20552076241277886.docx]

Anlage 16: Qualitative Interview Guideline

Let me shortly talk you through the consent form…

**Background**

- Can you tell me a bit about your education, experience with caring for people with dementia?
- Can you tell me about your thoughts on loneliness in people with dementia?
  - Importance in the care practice
  - Things that help
  - Things that make it worse
- *Loneliness is simply being away from people you would like to connect.*
- *Implementation is ‘group of processes intended to get an intervention into the use within an organisation’ (Nilsen & Bernhardsson, 2019).*

**Loneliness**

1. How is your overall impression with SilverFit Mile?
   - Regarding loneliness in people with dementia?
2. How well do you think the Mile addressed the loneliness in people with dementia?
3. How did the Mile compare to other alternatives for loneliness? (e.g. in house interventions such as pet therapy or videocalls with family).
4. Is there another alternative that you would rather implement for loneliness?
   - Can you describe that device?
   - Why would you prefer the alternative?
5. How essential is Mile to address loneliness?

**Implementation**

1. What was the initial reason behind getting a Mile in your care organization?
2. Were there any influencing factors such as policies or guidelines that impacted the decision to implement the Mile?
3. Were changes needed for Mile to work effectively in your care organization?
4. Were there any barriers to use with Mile e.g. complicated to set up or not suitable for everyone?
5. Were there any barriers for people with dementia face when using the Mile?
   - Can you tell me a story?
6. Were there any supports such as manuals or demonstrations by SilverFit to implement it?
7. How were the costs covered?
   - Was there any financial support?
8. In your opinion, is staff aware of the needs and preferences of the persons with dementia here?
   - Were their needs and preferences considered when deciding to get the Mile?
9. Do you know about any other organizations that have implemented the Mile for loneliness or other similar devices?
10. Were any infrastructure changes needed to implement Mile?
11. How well does Mile fit with your values and norms within the organization?
12. How well does Mile fit with existing work processes and practices in your setting?
13. Did Mile replaced a current program or process for loneliness?
    - In what ways?
14. Was there a plan to implement Mile?
    - Can you describe the plan for implementing Mile?
    - How did it start?
    - Was it implemented according to the implementation plan?
15. Did someone outside your organization helped you with implementing Mile?
16. Did you take any steps to encourage individuals to commit to using Mile?
    - Which individuals did you target?
    - How did you approach them?
17. Did you collect any information with Mile?
18. Did you assess progress towards implementation goals?
    - Was loneliness one of them?

**Thank you for your time!**
