# Supplementary material for: There is no one size fits all: Elements of implementing virtual bike rides to address loneliness in people living with dementia
Source: Digit Health. 2024 Sep 26;10:20552076241277886. doi: 10.1177/20552076241277886 (PMC11437561; doi:10.1177/20552076241277886)
Supplement: sj-docx-4-dhj-10.1177_20552076241277886 - Supplemental material for There is no one size fits all: Elements of implementing virtual bike rides to address loneliness in people living with dementia [file sj-docx-4-dhj-10.1177_20552076241277886.docx]

Anlage Nr. 4: Participant Information for qualitative interviews

**Technology for loneliness in dementia (TALenD)**

**Part of the EU project DISTINCT**

| ***Controller according to Article 4(7) GDPR:***  **Deutsches Zentrum für Neurodegenerative Erkrankungen e.V. (DZNE), Venusberg-Campus 1, Gebäude 99, 53127 Bonn.** |  |
| --- | --- |
| **Principal Investigator:**  DZNE e.V. – Location Witten  Prof. Dr. Martina Roes  Stockumer Str. 12  58453 Witten  Germany  Tel. no.: +49 2302 926 237  Email: martina.roes@dzne.de |  |

**Participant Information: Professional caregivers**

**Invitation to join the study TALenD: *Technology Alleviating Loneliness in Dementia***

Dear potential participant,

We would like to ask you about your willingness to participate in the above-mentioned study. Please read the following information carefully and ask Beliz Budak, the researcher, if you do not understand something.

The study is carried out by the German Centre for Neurodegenerative Diseases e.V. (DZNE) in Witten, Germany.

**Aim of the study**

The value of assistive technologies such as companion robots, like baby seal robot PARO in the care of persons living with dementia is well-recognized. They can positively impact various aspects of life, such as quality of life or loneliness. Some of the assistive technologies are known to be used to improve communication for people living with dementia. These technologies have different features and they can perform many different tasks, such as making it easier to communicate with family and friends or keeping people company. Assistive technologies are used to provide support with a number of challenges that can be observed frequently in persons living with dementia in long-term care environments, such as agitation or pain. However, there is a need for research on the implementation of those technologies.

**This research aims to identify aspects that can help or hinder the implementation of assistive technologies**. Specifically, we are interested in those technologies that might help to address loneliness in people with dementia living in long-term care. These interviews will be used to identify the factors affecting implementation of assistive technologies that are used to help addressing loneliness. We would like to understand how you think and feel about using assistive technologies in this context.

These interviews are a part of Beliz Budak’s PhD-studies and are a subproject of the EU-funded Marie Skłodowska-Curie project DISTINCT.

**Who can participate in the study?**

- Professional caregivers and/or managers of long-term care facilities
- Participants who speak English
- To participate in the study, you have to sign a form, giving consent to:
  - Willingness to participate in conversations with Beliz Budak and other staff for an hour and allowing Beliz to write this down in notes and record it in audio format for further examination.

**Methods/How does the study take place?**

The study will consist of an interview, which will take approximately 1 hour. You will be invited to an online meeting. You will be asked a few questions. The aim is to learn about your experiences and opinions on using assistive technology such as the baby seal robot Paro. Interviews will be digitally recorded with audio recording devices. The researcher then will analyse the data for an academic paper on implementation factors.

**Benefits and risks**

We do not expect that participation in the project will have any negative consequences for everyday life. There are no direct or indirect risks through participation. The investigator is an experienced researcher in this field. Furthermore, you will be acquiring knowledge on the topic and a copy of the article with the published findings from this study will be shared with you. You do not have a direct personal advantage by participating in this study.

**What happens to my data?**

During the study, personal information about you will be collected and recorded on electronic media (audio recording). The data that is stored, evaluated and if necessary, passed on in pseudonymised form (i.e. without mentioning your name or identifying data, but only with an assigned pseudonym) in the DZNE digital storage. Your identifying data (e.g. name, address, date of birth) are stored separately from the study data. Audio recordings will be deleted when the project ends, which is estimated to be in April 2023. The data will be deleted 10 years after the end of the study. Publications will not identify you as a participant unless you have agreed in your informed consent that you can be identified as a person in publications. You have the right to data transfer or information about the data processed by us about you. Please contact the persons below for further information. My data will be deleted 10 years after the end of the study. Audio recordings will be deleted when the project ends, which is estimated to be in April 2023. No conclusions about my person will be possible from scientific publications. Audio material that can be traced back to me will only be published if I have given my consent to the publication in a separate declaration of consent after the material is presented to me.

**How is my data protected?**

The information will be kept safe in terms of data protection. The data is secured against unauthorized access by means of encryption.

**Will I receive an expense allowance within the scope of participating in the study?**

No direct costs are expected to occur. Your time and involvement would be most appreciated but are not remunerable.

**My rights**

Participation in the survey is voluntary. You may revoke your participation and withdraw at any time without giving reasons and of course without incurring any disadvantages. At your request, we will correct, anonymise or delete your data. If the data has already been anonymised (the data can no longer be associated with you) or has already been used in analyses, deletion is no longer possible. You can object at any time to the processing of your data or demand a restriction on processing. You have the right to data transfer or information about the data processed by us about you. Please contact the persons below for further information.

**Data protection officer of the DZNE:**

Data protection officer

Deutsches Zentrum für Neurodegenerative Erkrankungen (DZNE)

Venusberg-Campus 1, Building 99

53127 Bonn

Tel no.: +49 228 433 020

Email: [datenschutz@dzne.de](mailto:datenschutz@dzne.de)

**Principal Investigator for this scientific study:**

Prof. Dr. Martina Roes

DZNE e.V. – Location Witten

Stockumer Straße 12

58453 Witten

Tel. no.: +49 2302 926 237

Email: [martina.roes@dzne.de](mailto:martina.roes@dzne.de)

Of course, you have the right to contact the responsible supervisory authority at any time.

**Address of the responsible supervisory authority:**

Federal Commissioner for Data Protection and Freedom of Information

Graurheindorfer Straße 153

53117 Bonn

Tel no.: +49 228 9977990

Email: [referat13@bfdi.bund.de](mailto:referat13@bfdi.bund.de)

**Your contact to the study team:**

DZNE e.V. – Location Witten

Beliz Budak

Stockumer Straße 12

58453 Witten

Tel. no: +49 23 02 - 926 231

Email: beliz.budak@dzne.de

**Who has reviewed this study?**

This study has received ethical approval from the Ethics Committee of the University Witten/Herdecke in Germany, approval number: SR-205/2021.

***Thank you for considering taking part in this study and
for taking time to read this invitation!***
